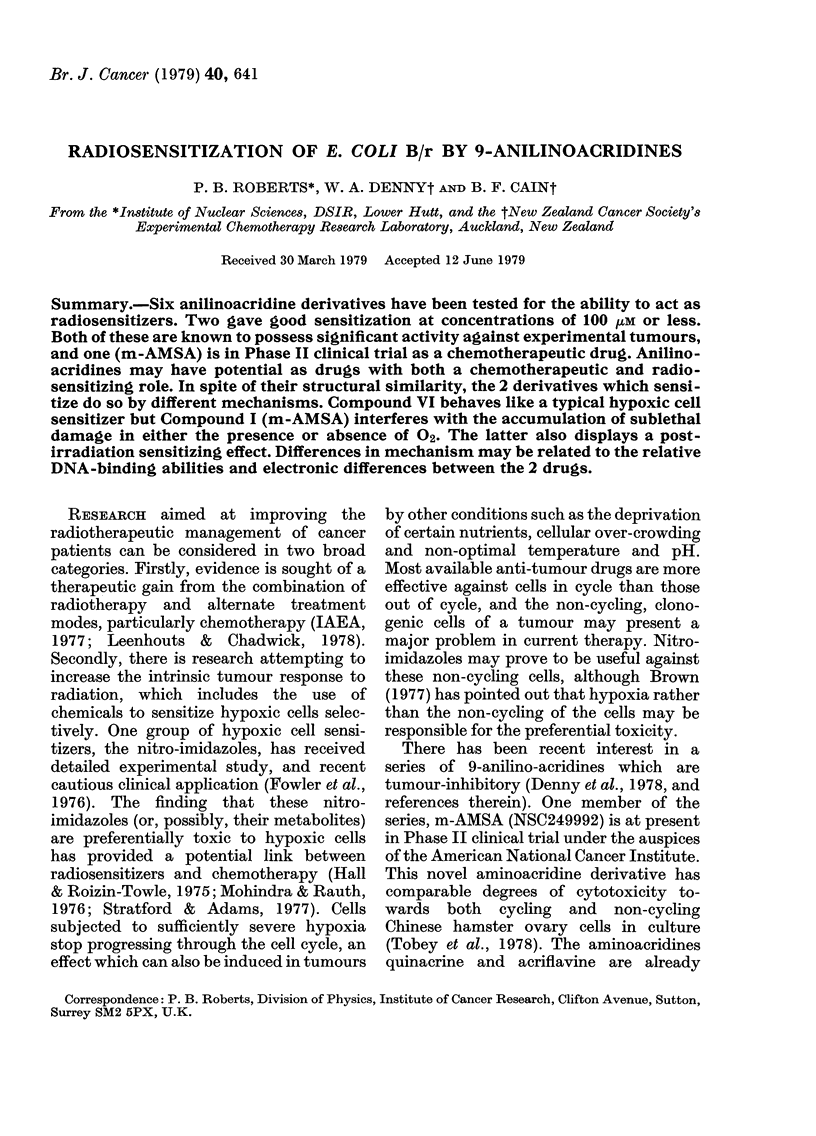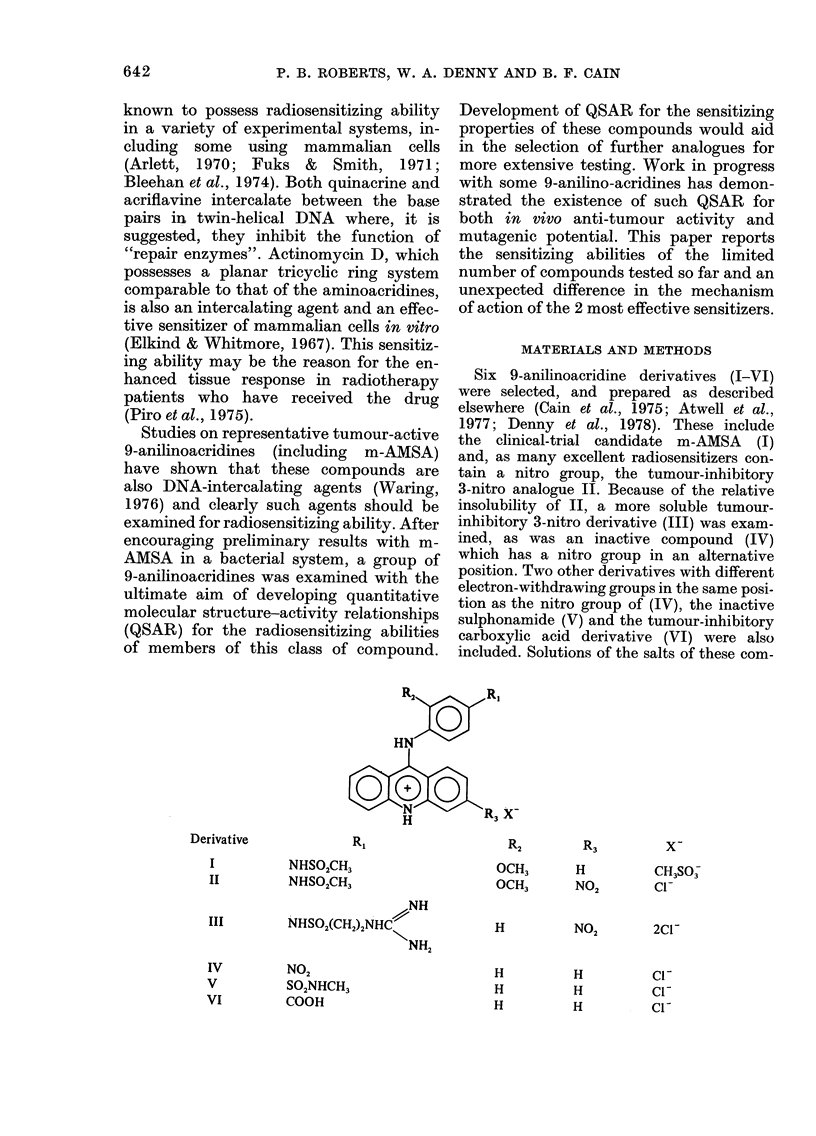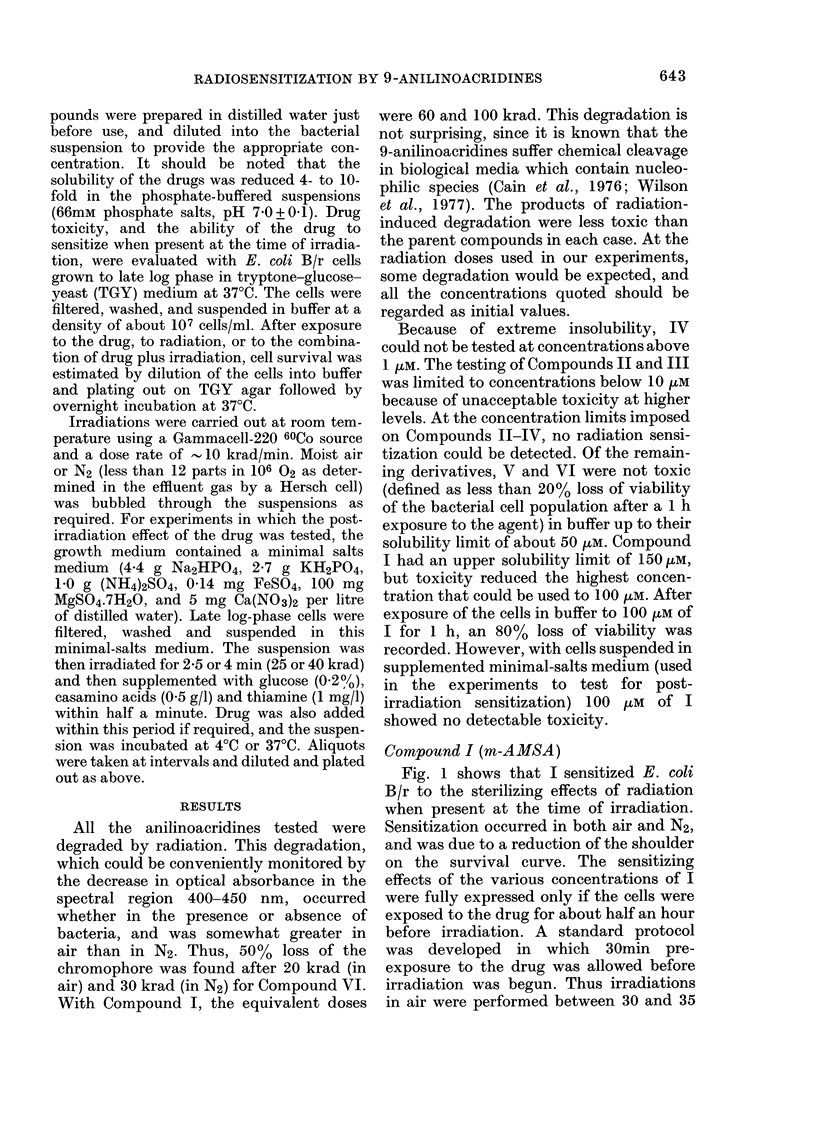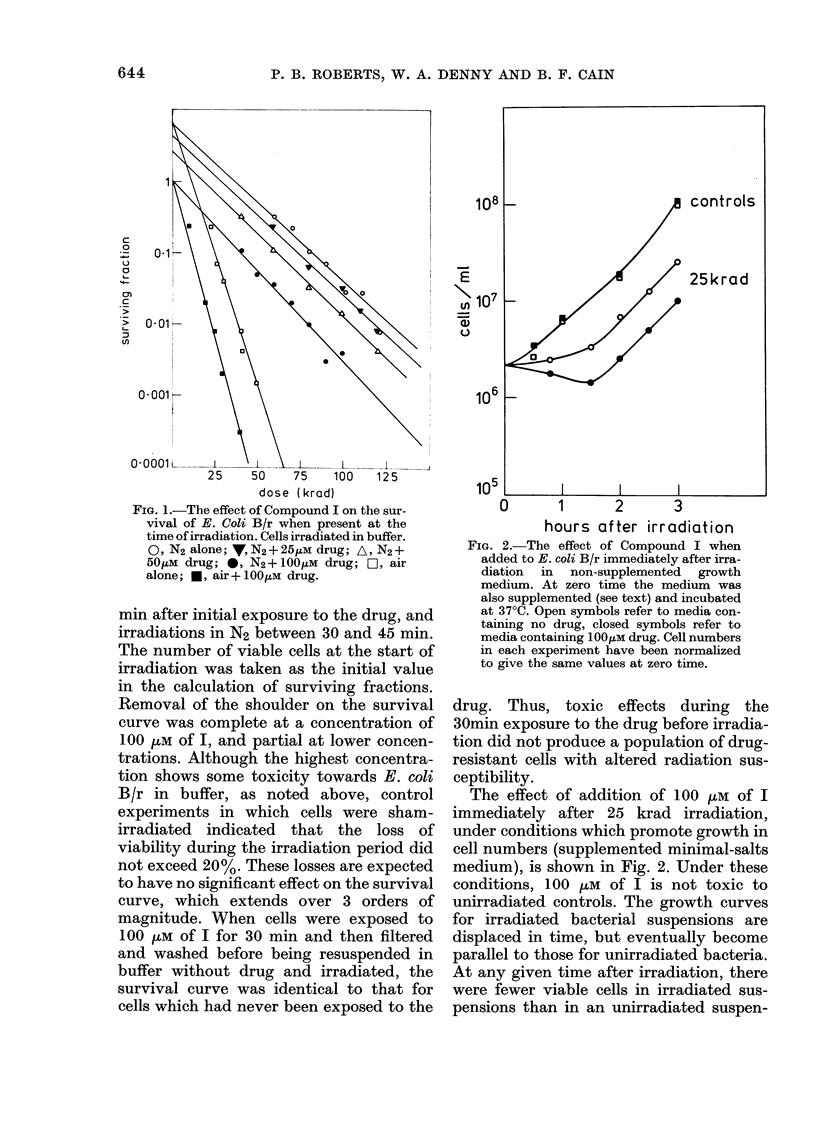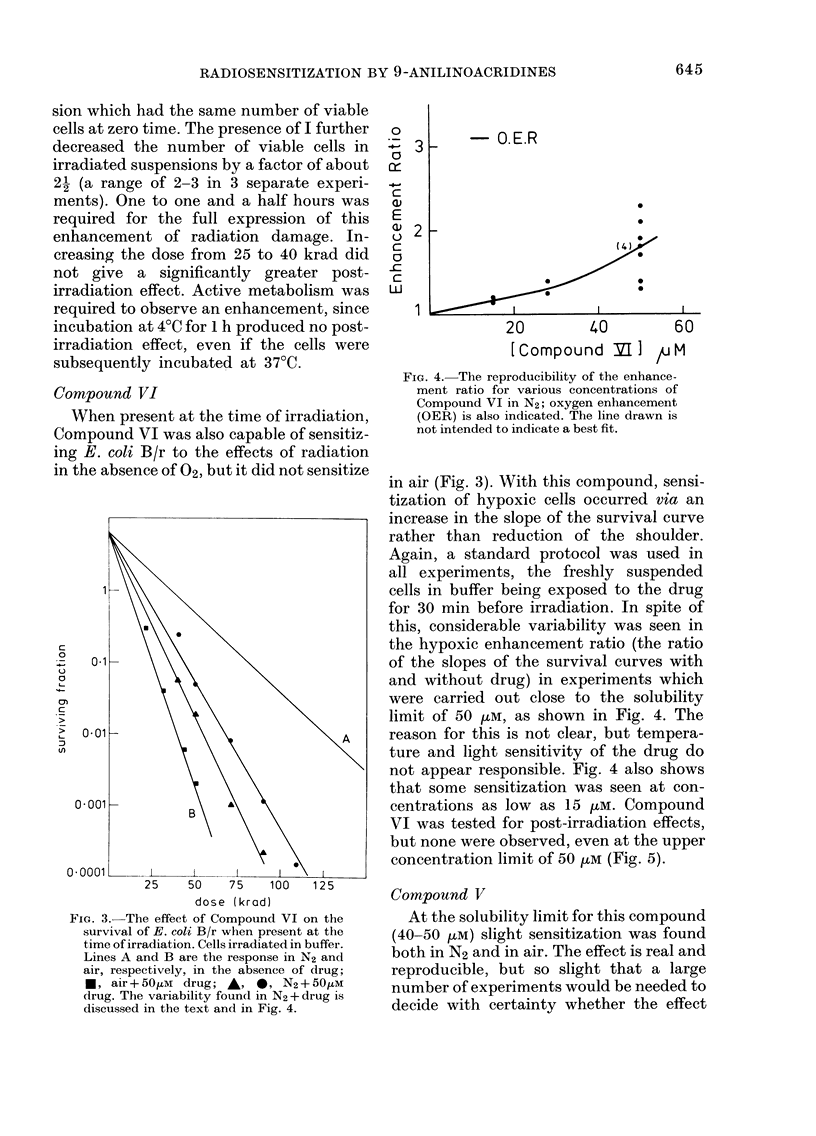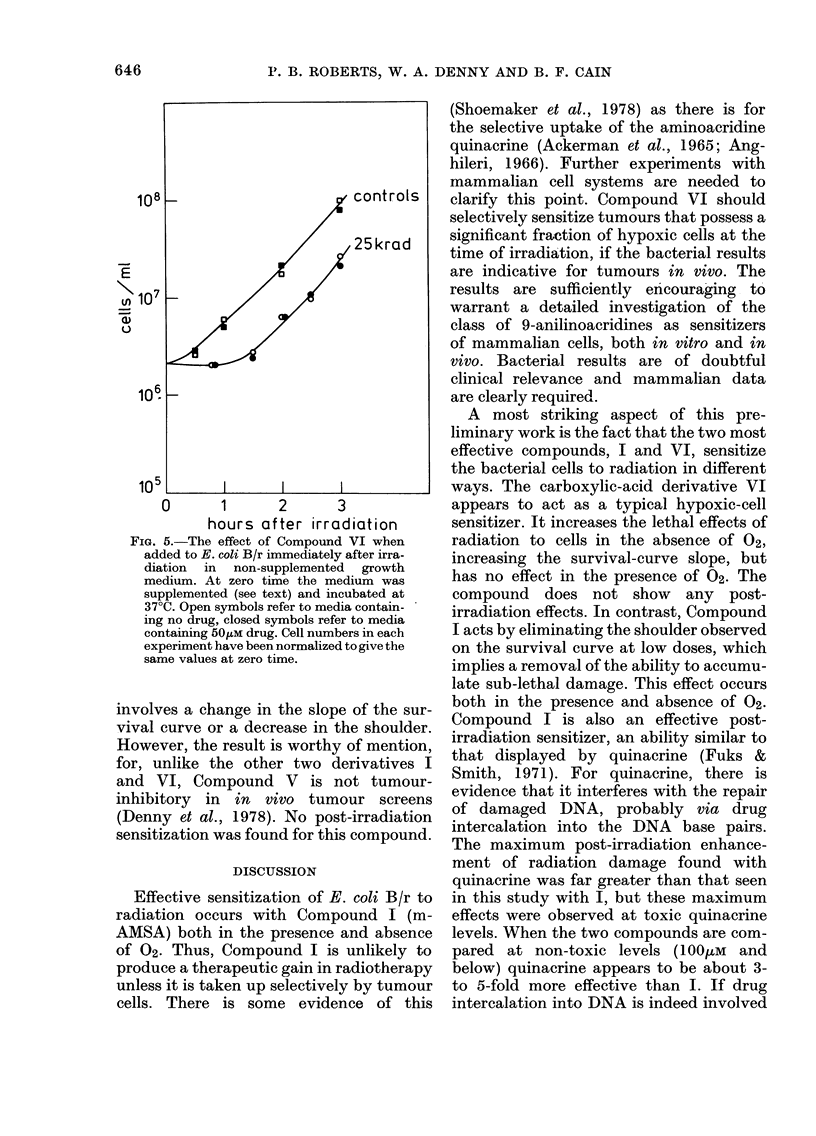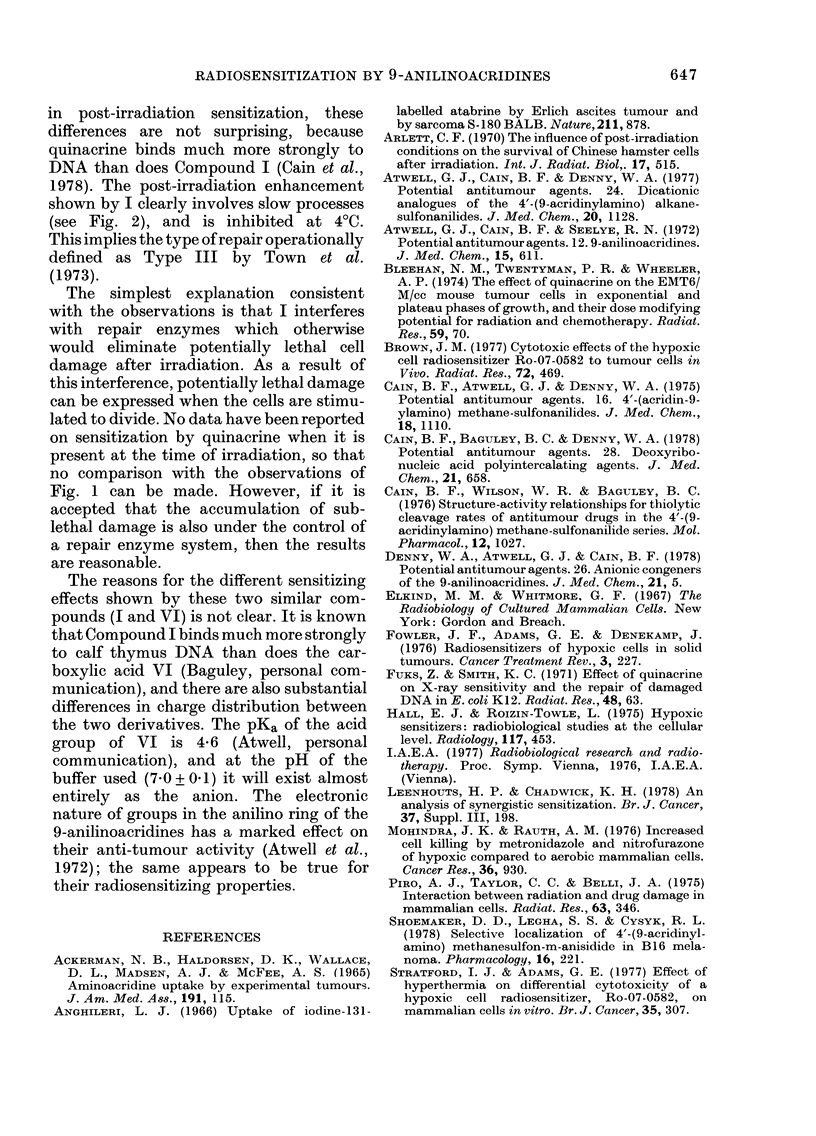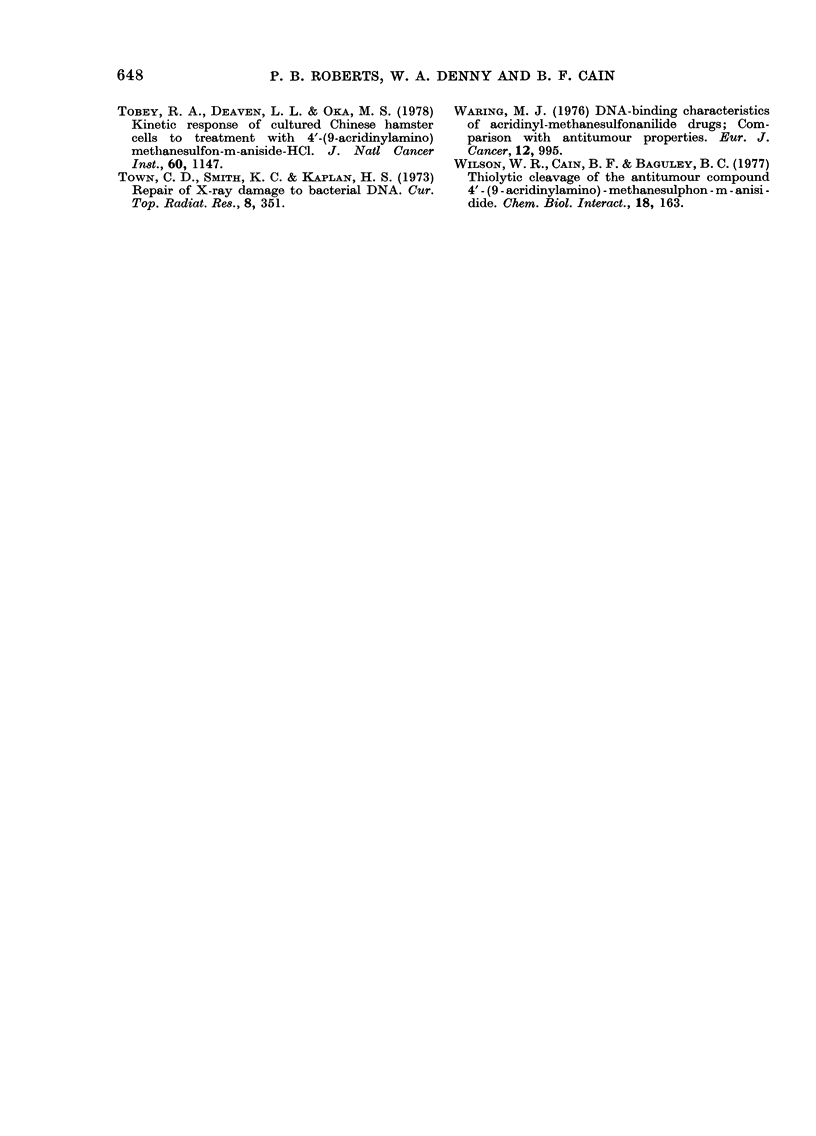# Radiosensitization of E. coli B/r by 9-anilinoacridines.

**DOI:** 10.1038/bjc.1979.230

**Published:** 1979-10

**Authors:** P. B. Roberts, W. A. Denny, B. F. Cain

## Abstract

Six anilinoacridine derivatives have been tested for the ability to act as radiosensitizers. Two gave good sensitization at concentrations of 100 microM or less. Both of these are known to possess significant activity against experimental tumours, and one (m-AMSA) is in Phase II clinical trial as a chemotherapeutic drug. Anilinoacridines may have potential as drugs with both a chemotherapeutic and radiosensitizing role. In spite of their structural similarity, the 2 derivatives which sensitize do so by different mechanisms. Compound VI behaves like a typical hypoxic cell sensitizer but Compound I (m-AMSA) interferes with the accumulation of sublethal damage in either the presence or absence of O2. The latter also displays a post-irradiation sensitizing effect. Differences in mechanism may be related to the relative DNA-binding abilities and electronic differences between the 2 drugs.


*Br. J. Cancer* (1979) **40**, 641

### RADIOSENSITIZATION OF *E. COLI* B/r BY 9-ANILINOACRIDINES

P. B. ROBERTS*, W. A. DENNY† AND B. F. CAIN†

*From the *Institute of Nuclear Sciences, DSIR, Lower Hutt, and the †New Zealand Cancer Society's Experimental Chemotherapy Research Laboratory, Auckland, New Zealand*

Received 30 March 1979 Accepted 12 June 1979

**Summary.—Six anilinoacridine derivatives have been tested for the ability to act as radiosensitizers. Two gave good sensitization at concentrations of 100 $\mu$M or less. Both of these are known to possess significant activity against experimental tumours, and one (m-AMSA) is in Phase II clinical trial as a chemotherapeutic drug. Anilinoacridines may have potential as drugs with both a chemotherapeutic and radiosensitizing role. In spite of their structural similarity, the 2 derivatives which sensitize do so by different mechanisms. Compound VI behaves like a typical hypoxic cell sensitizer but Compound I (m-AMSA) interferes with the accumulation of sublethal damage in either the presence or absence of $O_2$. The latter also displays a post-irradiation sensitizing effect. Differences in mechanism may be related to the relative DNA-binding abilities and electronic differences between the 2 drugs.**

RESEARCH aimed at improving the radiotherapeutic management of cancer patients can be considered in two broad categories. Firstly, evidence is sought of a therapeutic gain from the combination of radiotherapy and alternate treatment modes, particularly chemotherapy (IAEA, 1977; Leenhouts & Chadwick, 1978). Secondly, there is research attempting to increase the intrinsic tumour response to radiation, which includes the use of chemicals to sensitize hypoxic cells selectively. One group of hypoxic cell sensitizers, the nitro-imidazoles, has received detailed experimental study, and recent cautious clinical application (Fowler *et al.*, 1976). The finding that these nitro-imidazoles (or, possibly, their metabolites) are preferentially toxic to hypoxic cells has provided a potential link between radiosensitizers and chemotherapy (Hall & Roizin-Towle, 1975; Mohindra & Rauth, 1976; Stratford & Adams, 1977). Cells subjected to sufficiently severe hypoxia stop progressing through the cell cycle, an effect which can also be induced in tumours by other conditions such as the deprivation of certain nutrients, cellular over-crowding and non-optimal temperature and pH. Most available anti-tumour drugs are more effective against cells in cycle than those out of cycle, and the non-cycling, clonogenic cells of a tumour may present a major problem in current therapy. Nitro-imidazoles may prove to be useful against these non-cycling cells, although Brown (1977) has pointed out that hypoxia rather than the non-cycling of the cells may be responsible for the preferential toxicity.

There has been recent interest in a series of 9-anilino-acridines which are tumour-inhibitory (Denny *et al.*, 1978, and references therein). One member of the series, m-AMSA (NSC249992) is at present in Phase II clinical trial under the auspices of the American National Cancer Institute. This novel aminoacridine derivative has comparable degrees of cytotoxicity towards both cycling and non-cycling Chinese hamster ovary cells in culture (Tobey *et al.*, 1978). The aminoacridines quinacrine and acriflavine are already

Correspondence: P. B. Roberts, Division of Physics, Institute of Cancer Research, Clifton Avenue, Sutton, Surrey SM2 5PX, U.K.

known to possess radiosensitizing ability in a variety of experimental systems, including some using mammalian cells (Arlett, 1970; Fuks & Smith, 1971; Bleehan *et al.*, 1974). Both quinacrine and acriflavine intercalate between the base pairs in twin-helical DNA where, it is suggested, they inhibit the function of "repair enzymes". Actinomycin D, which possesses a planar tricyclic ring system comparable to that of the aminoacridines, is also an intercalating agent and an effective sensitizer of mammalian cells *in vitro* (Elkind & Whitmore, 1967). This sensitizing ability may be the reason for the enhanced tissue response in radiotherapy patients who have received the drug (Piro *et al.*, 1975).

Studies on representative tumour-active 9-anilinoacridines (including m-AMSA) have shown that these compounds are also DNA-intercalating agents (Waring, 1976) and clearly such agents should be examined for radiosensitizing ability. After encouraging preliminary results with m-AMSA in a bacterial system, a group of 9-anilinoacridines was examined with the ultimate aim of developing quantitative molecular structure–activity relationships (QSAR) for the radiosensitizing abilities of members of this class of compound. Development of QSAR for the sensitizing properties of these compounds would aid in the selection of further analogues for more extensive testing. Work in progress with some 9-anilino-acridines has demonstrated the existence of such QSAR for both *in vivo* anti-tumour activity and mutagenic potential. This paper reports the sensitizing abilities of the limited number of compounds tested so far and an unexpected difference in the mechanism of action of the 2 most effective sensitizers.

### MATERIALS AND METHODS

Six 9-anilinoacridine derivatives (I–VI) were selected, and prepared as described elsewhere (Cain *et al.*, 1975; Atwell *et al.*, 1977; Denny *et al.*, 1978). These include the clinical-trial candidate m-AMSA (I) and, as many excellent radiosensitizers contain a nitro group, the tumour-inhibitory 3-nitro analogue II. Because of the relative insolubility of II, a more soluble tumour-inhibitory 3-nitro derivative (III) was examined, as was an inactive compound (IV) which has a nitro group in an alternative position. Two other derivatives with different electron-withdrawing groups in the same position as the nitro group of (IV), the inactive sulphonamide (V) and the tumour-inhibitory carboxylic acid derivative (VI) were also included. Solutions of the salts of these com-

| Derivative | $R_1$ | $R_2$ | $R_3$ | $X^-$ |
|---|---|---|---|---|
| I | $NHSO_2CH_3$ | $OCH_3$ | H | $CH_3SO_3^-$ |
| II | $NHSO_2CH_3$ | $OCH_3$ | $NO_2$ | $Cl^-$ |
| III | $NHSO_2(CH_2)_2NHC(=NH)NH_2$ | H | $NO_2$ | $2Cl^-$ |
| IV | $NO_2$ | H | H | $Cl^-$ |
| V | $SO_2NHCH_3$ | H | H | $Cl^-$ |
| VI | COOH | H | H | $Cl^-$ |

pounds were prepared in distilled water just before use, and diluted into the bacterial suspension to provide the appropriate concentration. It should be noted that the solubility of the drugs was reduced 4- to 10-fold in the phosphate-buffered suspensions (66mM phosphate salts, pH $7{\cdot}0 \pm 0{\cdot}1$). Drug toxicity, and the ability of the drug to sensitize when present at the time of irradiation, were evaluated with *E. coli* B/r cells grown to late log phase in tryptone–glucose–yeast (TGY) medium at 37°C. The cells were filtered, washed, and suspended in buffer at a density of about $10^7$ cells/ml. After exposure to the drug, to radiation, or to the combination of drug plus irradiation, cell survival was estimated by dilution of the cells into buffer and plating out on TGY agar followed by overnight incubation at 37°C.

Irradiations were carried out at room temperature using a Gammacell-220 $^{60}Co$ source and a dose rate of $\sim$10 krad/min. Moist air or $N_2$ (less than 12 parts in $10^6$ $O_2$ as determined in the effluent gas by a Hersch cell) was bubbled through the suspensions as required. For experiments in which the post-irradiation effect of the drug was tested, the growth medium contained a minimal salts medium (4·4 g $Na_2HPO_4$, 2·7 g $KH_2PO_4$, 1·0 g $(NH_4)_2SO_4$, 0·14 mg $FeSO_4$, 100 mg $MgSO_4.7H_2O$, and 5 mg $Ca(NO_3)_2$ per litre of distilled water). Late log-phase cells were filtered, washed and suspended in this minimal-salts medium. The suspension was then irradiated for 2·5 or 4 min (25 or 40 krad) and then supplemented with glucose (0·2%), casamino acids (0·5 g/l) and thiamine (1 mg/l) within half a minute. Drug was also added within this period if required, and the suspension was incubated at 4°C or 37°C. Aliquots were taken at intervals and diluted and plated out as above.

### RESULTS

All the anilinoacridines tested were degraded by radiation. This degradation, which could be conveniently monitored by the decrease in optical absorbance in the spectral region 400–450 nm, occurred whether in the presence or absence of bacteria, and was somewhat greater in air than in $N_2$. Thus, 50% loss of the chromophore was found after 20 krad (in air) and 30 krad (in $N_2$) for Compound VI. With Compound I, the equivalent doses were 60 and 100 krad. This degradation is not surprising, since it is known that the 9-anilinoacridines suffer chemical cleavage in biological media which contain nucleophilic species (Cain *et al.*, 1976; Wilson *et al.*, 1977). The products of radiation-induced degradation were less toxic than the parent compounds in each case. At the radiation doses used in our experiments, some degradation would be expected, and all the concentrations quoted should be regarded as initial values.

Because of extreme insolubility, IV could not be tested at concentrations above 1 $\mu$M. The testing of Compounds II and III was limited to concentrations below 10 $\mu$M because of unacceptable toxicity at higher levels. At the concentration limits imposed on Compounds II–IV, no radiation sensitization could be detected. Of the remaining derivatives, V and VI were not toxic (defined as less than 20% loss of viability of the bacterial cell population after a 1 h exposure to the agent) in buffer up to their solubility limit of about 50 $\mu$M. Compound I had an upper solubility limit of 150 $\mu$M, but toxicity reduced the highest concentration that could be used to 100 $\mu$M. After exposure of the cells in buffer to 100 $\mu$M of I for 1 h, an 80% loss of viability was recorded. However, with cells suspended in supplemented minimal-salts medium (used in the experiments to test for post-irradiation sensitization) 100 $\mu$M of I showed no detectable toxicity.

### *Compound I (m-AMSA)*

Fig. 1 shows that I sensitized *E. coli* B/r to the sterilizing effects of radiation when present at the time of irradiation. Sensitization occurred in both air and $N_2$, and was due to a reduction of the shoulder on the survival curve. The sensitizing effects of the various concentrations of I were fully expressed only if the cells were exposed to the drug for about half an hour before irradiation. A standard protocol was developed in which 30min pre-exposure to the drug was allowed before irradiation was begun. Thus irradiations in air were performed between 30 and 35

FIG. 1.—The effect of Compound I on the survival of *E. Coli* B/r when present at the time of irradiation. Cells irradiated in buffer. ○, $N_2$ alone; ▼, $N_2$ + 25μM drug; △, $N_2$ + 50μM drug; ●, $N_2$ + 100μM drug; □, air alone; ■, air + 100μM drug.

FIG. 2.—The effect of Compound I when added to *E. coli* B/r immediately after irradiation in non-supplemented growth medium. At zero time the medium was also supplemented (see text) and incubated at 37°C. Open symbols refer to media containing no drug, closed symbols refer to media containing 100μM drug. Cell numbers in each experiment have been normalized to give the same values at zero time.

min after initial exposure to the drug, and irradiations in $N_2$ between 30 and 45 min. The number of viable cells at the start of irradiation was taken as the initial value in the calculation of surviving fractions. Removal of the shoulder on the survival curve was complete at a concentration of 100 μM of I, and partial at lower concentrations. Although the highest concentration shows some toxicity towards *E. coli* B/r in buffer, as noted above, control experiments in which cells were sham-irradiated indicated that the loss of viability during the irradiation period did not exceed 20%. These losses are expected to have no significant effect on the survival curve, which extends over 3 orders of magnitude. When cells were exposed to 100 μM of I for 30 min and then filtered and washed before being resuspended in buffer without drug and irradiated, the survival curve was identical to that for cells which had never been exposed to the drug. Thus, toxic effects during the 30min exposure to the drug before irradiation did not produce a population of drug-resistant cells with altered radiation susceptibility.

The effect of addition of 100 μM of I immediately after 25 krad irradiation, under conditions which promote growth in cell numbers (supplemented minimal-salts medium), is shown in Fig. 2. Under these conditions, 100 μM of I is not toxic to unirradiated controls. The growth curves for irradiated bacterial suspensions are displaced in time, but eventually become parallel to those for unirradiated bacteria. At any given time after irradiation, there were fewer viable cells in irradiated suspensions than in an unirradiated suspension which had the same number of viable cells at zero time. The presence of I further decreased the number of viable cells in irradiated suspensions by a factor of about 2½ (a range of 2–3 in 3 separate experiments). One to one and a half hours was required for the full expression of this enhancement of radiation damage. Increasing the dose from 25 to 40 krad did not give a significantly greater post-irradiation effect. Active metabolism was required to observe an enhancement, since incubation at 4°C for 1 h produced no post-irradiation effect, even if the cells were subsequently incubated at 37°C.

*Compound VI*

When present at the time of irradiation, Compound VI was also capable of sensitizing *E. coli* B/r to the effects of radiation in the absence of $O_2$, but it did not sensitize in air (Fig. 3). With this compound, sensitization of hypoxic cells occurred *via* an increase in the slope of the survival curve rather than reduction of the shoulder. Again, a standard protocol was used in all experiments, the freshly suspended cells in buffer being exposed to the drug for 30 min before irradiation. In spite of this, considerable variability was seen in the hypoxic enhancement ratio (the ratio of the slopes of the survival curves with and without drug) in experiments which were carried out close to the solubility limit of 50 μM, as shown in Fig. 4. The reason for this is not clear, but temperature and light sensitivity of the drug do not appear responsible. Fig. 4 also shows that some sensitization was seen at concentrations as low as 15 μM. Compound VI was tested for post-irradiation effects, but none were observed, even at the upper concentration limit of 50 μM (Fig. 5).

FIG. 4.—The reproducibility of the enhancement ratio for various concentrations of Compound VI in $N_2$; oxygen enhancement (OER) is also indicated. The line drawn is not intended to indicate a best fit.

FIG. 3.—The effect of Compound VI on the survival of *E. coli* B/r when present at the time of irradiation. Cells irradiated in buffer. Lines A and B are the response in $N_2$ and air, respectively, in the absence of drug; ■, air + 50μM drug; ▲, ●, $N_2$ + 50μM drug. The variability found in $N_2$ + drug is discussed in the text and in Fig. 4.

*Compound V*

At the solubility limit for this compound (40–50 μM) slight sensitization was found both in $N_2$ and in air. The effect is real and reproducible, but so slight that a large number of experiments would be needed to decide with certainty whether the effect

FIG. 5.—The effect of Compound VI when added to *E. coli* B/r immediately after irradiation in non-supplemented growth medium. At zero time the medium was supplemented (see text) and incubated at 37°C. Open symbols refer to media containing no drug, closed symbols refer to media containing 50$\mu$M drug. Cell numbers in each experiment have been normalized to give the same values at zero time.

involves a change in the slope of the survival curve or a decrease in the shoulder. However, the result is worthy of mention, for, unlike the other two derivatives I and VI, Compound V is not tumour-inhibitory in *in vivo* tumour screens (Denny *et al.*, 1978). No post-irradiation sensitization was found for this compound.

### DISCUSSION

Effective sensitization of *E. coli* B/r to radiation occurs with Compound I (m-AMSA) both in the presence and absence of $O_2$. Thus, Compound I is unlikely to produce a therapeutic gain in radiotherapy unless it is taken up selectively by tumour cells. There is some evidence of this (Shoemaker *et al.*, 1978) as there is for the selective uptake of the aminoacridine quinacrine (Ackerman *et al.*, 1965; Anghileri, 1966). Further experiments with mammalian cell systems are needed to clarify this point. Compound VI should selectively sensitize tumours that possess a significant fraction of hypoxic cells at the time of irradiation, if the bacterial results are indicative for tumours *in vivo*. The results are sufficiently encouraging to warrant a detailed investigation of the class of 9-anilinoacridines as sensitizers of mammalian cells, both *in vitro* and *in vivo*. Bacterial results are of doubtful clinical relevance and mammalian data are clearly required.

A most striking aspect of this preliminary work is the fact that the two most effective compounds, I and VI, sensitize the bacterial cells to radiation in different ways. The carboxylic-acid derivative VI appears to act as a typical hypoxic-cell sensitizer. It increases the lethal effects of radiation to cells in the absence of $O_2$, increasing the survival-curve slope, but has no effect in the presence of $O_2$. The compound does not show any post-irradiation effects. In contrast, Compound I acts by eliminating the shoulder observed on the survival curve at low doses, which implies a removal of the ability to accumulate sub-lethal damage. This effect occurs both in the presence and absence of $O_2$. Compound I is also an effective post-irradiation sensitizer, an ability similar to that displayed by quinacrine (Fuks & Smith, 1971). For quinacrine, there is evidence that it interferes with the repair of damaged DNA, probably *via* drug intercalation into the DNA base pairs. The maximum post-irradiation enhancement of radiation damage found with quinacrine was far greater than that seen in this study with I, but these maximum effects were observed at toxic quinacrine levels. When the two compounds are compared at non-toxic levels (100$\mu$M and below) quinacrine appears to be about 3- to 5-fold more effective than I. If drug intercalation into DNA is indeed involved in post-irradiation sensitization, these differences are not surprising, because quinacrine binds much more strongly to DNA than does Compound I (Cain *et al.*, 1978). The post-irradiation enhancement shown by I clearly involves slow processes (see Fig. 2), and is inhibited at 4°C. This implies the type of repair operationally defined as Type III by Town *et al.* (1973).

The simplest explanation consistent with the observations is that I interferes with repair enzymes which otherwise would eliminate potentially lethal cell damage after irradiation. As a result of this interference, potentially lethal damage can be expressed when the cells are stimulated to divide. No data have been reported on sensitization by quinacrine when it is present at the time of irradiation, so that no comparison with the observations of Fig. 1 can be made. However, if it is accepted that the accumulation of sublethal damage is also under the control of a repair enzyme system, then the results are reasonable.

The reasons for the different sensitizing effects shown by these two similar compounds (I and VI) is not clear. It is known that Compound I binds much more strongly to calf thymus DNA than does the carboxylic acid VI (Baguley, personal communication), and there are also substantial differences in charge distribution between the two derivatives. The $pK_a$ of the acid group of VI is 4·6 (Atwell, personal communication), and at the pH of the buffer used ($7{\cdot}0 \pm 0{\cdot}1$) it will exist almost entirely as the anion. The electronic nature of groups in the anilino ring of the 9-anilinoacridines has a marked effect on their anti-tumour activity (Atwell *et al.*, 1972); the same appears to be true for their radiosensitizing properties.

### REFERENCES

Ackerman, N. B., Haldorsen, D. K., Wallace, D. L., Madsen, A. J. & McFee, A. S. (1965) Aminoacridine uptake by experimental tumours. *J. Am. Med. Ass.*, **191**, 115.

Anghileri, L. J. (1966) Uptake of iodine-131-labelled atabrine by Erlich ascites tumour and by sarcoma S-180 BALB. *Nature*, **211**, 878.

Arlett, C. F. (1970) The influence of post-irradiation conditions on the survival of Chinese hamster cells after irradiation. *Int. J. Radiat. Biol,*. **17**, 515.

Atwell, G. J., Cain, B. F. & Denny, W. A. (1977) Potential antitumour agents. 24. Dicationic analogues of the 4′-(9-acridinylamino) alkanesulfonanilides. *J. Med. Chem.*, **20**, 1128.

Atwell, G. J., Cain, B. F. & Seelye, R. N. (1972) Potential antitumour agents. 12. 9-anilinoacridines. *J. Med. Chem.*, **15**, 611.

Bleehan, N. M., Twentyman, P. R. & Wheeler, A. P. (1974) The effect of quinacrine on the EMT6/M/cc mouse tumour cells in exponential and plateau phases of growth, and their dose modifying potential for radiation and chemotherapy. *Radiat. Res.*, **59**, 70.

Brown, J. M. (1977) Cytotoxic effects of the hypoxic cell radiosensitizer Ro-07-0582 to tumour cells *in Vivo. Radiat. Res.*, **72**, 469.

Cain, B. F., Atwell, G. J. & Denny, W. A. (1975) Potential antitumour agents. 16. 4′-(acridin-9-ylamino) methane-sulfonanilides. *J. Med. Chem.*, **18**, 1110.

Cain, B. F., Baguley, B. C. & Denny, W. A. (1978) Potential antitumour agents. 28. Deoxyribonucleic acid polyintercalating agents. *J. Med. Chem.*, **21**, 658.

Cain, B. F., Wilson, W. R. & Baguley, B. C. (1976) Structure-activity relationships for thiolytic cleavage rates of antitumour drugs in the 4′-(9-acridinylamino) methane-sulfonanilide series. *Mol. Pharmacol.*, **12**, 1027.

Denny, W. A., Atwell, G. J. & Cain, B. F. (1978) Potential antitumour agents. 26. Anionic congeners of the 9-anilinoacridines. *J. Med. Chem.*, **21**, 5.

Elkind, M. M. & Whitmore, G. F. (1967) *The Radiobiology of Cultured Mammalian Cells.* New York: Gordon and Breach.

Fowler, J. F., Adams, G. E. & Denekamp, J. (1976) Radiosensitizers of hypoxic cells in solid tumours. *Cancer Treatment Rev.*, **3**, 227.

Fuks, Z. & Smith, K. C. (1971) Effect of quinacrine on X-ray sensitivity and the repair of damaged DNA in *E. coli* K12. *Radiat. Res.*, **48**, 63.

Hall, E. J. & Roizin-Towle, L. (1975) Hypoxic sensitizers: radiobiological studies at the cellular level. *Radiology*, **117**, 453.

I.A.E.A. (1977) *Radiobiological research and radiotherapy.* Proc. Symp. Vienna, 1976, I.A.E.A. (Vienna).

Leenhouts, H. P. & Chadwick, K. H. (1978) An analysis of synergistic sensitization. *Br. J. Cancer*, **37**, Suppl. III, 198.

Mohindra, J. K. & Rauth, A. M. (1976) Increased cell killing by metronidazole and nitrofurazone of hypoxic compared to aerobic mammalian cells. *Cancer Res.*, **36**, 930.

Piro, A. J., Taylor, C. C. & Belli, J. A. (1975) Interaction between radiation and drug damage in mammalian cells. *Radiat. Res.*, **63**, 346.

Shoemaker, D. D., Legha, S. S. & Cysyk, R. L. (1978) Selective localization of 4′-(9-acridinylamino) methanesulfon-m-anisidide in B16 melanoma. *Pharmacology*, **16**, 221.

Stratford, I. J. & Adams, G. E. (1977) Effect of hyperthermia on differential cytotoxicity of a hypoxic cell radiosensitizer, Ro-07-0582, on mammalian cells *in vitro*. *Br. J. Cancer*, **35**, 307.

TOBEY, R. A., DEAVEN, L. L. & OKA, M. S. (1978) Kinetic response of cultured Chinese hamster cells to treatment with 4′-(9-acridinylamino) methanesulfon-m-aniside-HCl. *J. Natl Cancer Inst.*, **60**, 1147.

TOWN, C. D., SMITH, K. C. & KAPLAN, H. S. (1973) Repair of X-ray damage to bacterial DNA. *Cur. Top. Radiat. Res.*, **8**, 351.

WARING, M. J. (1976) DNA-binding characteristics of acridinyl-methanesulfonanilide drugs; Comparison with antitumour properties. *Eur. J. Cancer*, **12**, 995.

WILSON, W. R., CAIN, B. F. & BAGULEY, B. C. (1977) Thiolytic cleavage of the antitumour compound 4′-(9-acridinylamino)-methanesulphon-m-anisidide. *Chem. Biol. Interact.*, **18**, 163.